# Brazilian Amazon Traditional Medicine and the Treatment of Difficult to Heal Leishmaniasis Wounds with* Copaifera*

**DOI:** 10.1155/2017/8350320

**Published:** 2017-01-17

**Authors:** Kelly Cristina Oliveira de Albuquerque, Andreza do Socorro Silva da Veiga, João Victor da Silva e Silva, Heliton Patrick Cordovil Brigido, Erica Patrícia dos Reis Ferreira, Erica Vanessa Souza Costa, Andrey Moacir do Rosário Marinho, Sandro Percário, Maria Fâni Dolabela

**Affiliations:** ^1^Programa de Pós-Graduação em Ciências Farmacêuticas, Instituto de Ciências da Saúde, Universidade Federal do Pará, Belém, PA, Brazil; ^2^Programa de Pós-Graduação em Inovação Farmacêutica, Instituto de Ciências da Saúde, Universidade Federal do Pará, Belém, PA, Brazil; ^3^Faculdade de Química, Instituto de Ciências Exatas e Naturais, Universidade Federal do Pará, Belém, PA, Brazil; ^4^Laboratório de Estresse Oxidativo, Instituto de Ciências Biológicas, Universidade Federal do Pará, Belém, PA, Brazil

## Abstract

The present study describes the use of the traditional species* Copaifera* for treating wounds, such as ulcers scarring and antileishmanial wounds. It also relates phytochemical studies, evaluation of the leishmanicidal activity, and toxicity. The species of* Copaifera* with a higher incidence in the Amazon region are* Copaifera officinalis*,* Copaifera reticulata*,* Copaifera multijuga *Hayne. The copaiba oil is used in the Amazon's traditional medicine, especially as anti-inflammatory ingredient, in ulcers healing, and in scarring and for leishmaniasis. Chemical studies have shown that these oils contain diterpenes and sesquiterpenes. The copaiba oil and terpenes isolated have antiparasitic activity, more promising in the amastigote form of* L. amazonensis*. This activity is probably related to changes in the cell membrane and mitochondria. The oil showed low cytotoxicity and genotoxicity. Furthermore, it may interfere with immune response to infection and also has a healing effect. In summary, the copaiba oil is promising as leishmanicidal agent.

## 1. Introduction

In history, many civilizations have left traces of their culture on objects; moreover there were also expressed diseases that affected them. Skin lesions and human facial deformities were depicted in ceramics of Peru and Ecuador's pre-Inca civilizations; these ceramics are dated from the beginning of 1st century B.C. In the 15th and 16th centuries, texts found in the Americas mention the risk of getting the “diseases of the Andes”; these diseases afflict agricultural workers and are characterized by very debilitating injuries [1, 2].

In Brazil, the document of the Geographic Political Religious Pastoral (1827) is considered the first report of leishmaniasis in the country. This work recounts the journey of Don Frei Hipólito Sanches Fayas and Quiros Tabatinga (AM) by the Brazilian Amazon to Peru [3]. Moreira (1895) was the first to identify cases of leishmania in Brazil; this period was known as “Bahia's button” or “Biskra's Button.” Gaspar Viana (1911) identified the parasite involved in the pathology and named it as* Leishmania braziliensis* [4]. Aragon (1922) demonstrated the role of the sandfly in the transmission of cutaneous leishmaniasis. Since then, the transmission of the disease has been described in several cities of all federal units (UF) [5]. The cutaneous form is characterized by the presence of a difficult to heal “ulcer” [6].

Over the centuries, products of plant origin were the basis for treatment of various diseases. Phytochemical studies of some species led to the isolation of many drugs [7]. In the process of wound healing, their use is not different. Plants or extracts were used in the form of poultices to stop bleeding and promote healing [8]. The vegetable oil was used to promote healing [9] and the antileishmania activity [10].

After an extensive review of the literature, we noted the importance of the species* Copaifera* in the treatment of wounds and leishmaniasis [18, 19].* Copaifera* comprises nearly 72 species [13], mainly by their economic and medicinal importance [20, 21].

In the Americas 28 different species were described; in Brazil 16 of these species were identified [13]. The most abundant ones were* Copaifera officinalis *L. (North Amazonas, Roraima, Colombia, and Venezuela),* Copaifera guyanensis *Desf. (Guianas),* Copaifera reticulata *Ducke,* Copaifera multijuga *Hayne (Amazon),* Copaifera confertiflora *Benth. (Piauí),* Copaifera langsdorffii *Desf. (Brazil, Argentina, and Paraguay),* Copaifera coriacea *Mart. (Bahia) and* Copaifera cearensis *Huber ex Ducke (Ceará) [22–24].

Copaiba oil is used in the Amazon traditional medicine, especially as an anti-inflammatory ingredient and for wound healing [13, 25, 26], and its use has been reported since the 16th century. America's settlers reported that the American Indians applied this oil in the navel of newborns and wounds of the warriors after battles. This indigenous use originated from the observation of animals that, when wounded, rubbed themselves on the trunk of the copaiba tree to heal their wounds. [27]. In summary, exposure to copaiba oil accelerated the healing of wounds of different origins. The leishmanicidal activity has also been described [10].

**Figure 1:**
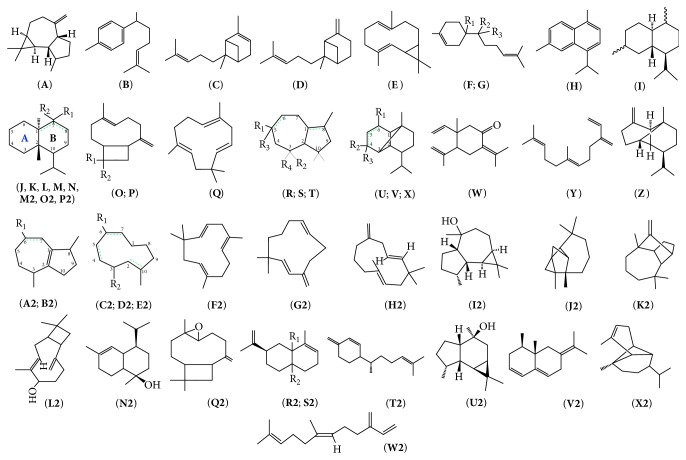
Sesquiterpenes found in copaiba oils. (**A**) Alloaromadendrene; (**B**) ar-curcumene; (**C**) *α*-bergamotene; (**D**) *β*-bergamotene; (**E**) bicyclogermacrene; (**F**) *β*-bisabolene [R_1_ = H, R_2_ and R_3_ = CH_2_]; (**G**) *β*-bisabolol** [**R_1_ = OH, R_2_ = CH_3_, R_3_ = H]; (**H**) cadalene; (**I**) cadinene; (**J**) *α*-cadinene [R_1_ = CH_3_; R_2_ = not; C_7_ = C_8_; A = 4- CH_3_-hexcycl-3-ene]; (**K**) *γ*-cadinene [R_1_ and R_2_ = CH_2_; C_7_ = C_8_; A = 4- CH_3_-hexcycl-3-ene]; (**L**) *δ*-cadinene [R_1_ = CH_3_; R_2_ = not; C_1_ = C_7_; A- CH_3_ -hexcycl- 3-ene]; (**M**) *α*-cadinol [R_1_ = H; R_2_ = OH; A = 4- CH_3_ -hexcycl-3-ene]; (**N**) calamenene [R_1_ = H; R_2_ = CH_3_; A = benzene]; (**O**) caryophyllene [R_1_ = CH_3_, R_2_ = CH_3_,* cis*]; (**P**) *β*-caryophyllene [R_1_ = CH_3_; R_2_ = CH_3_,* trans*]; (**Q**) *α*-caryophyllenol; (**R**) cedrol [R_1_ = H; R_2_ = CH_3_; R_3_ = OH; R_4_ = CH_3_; C_1_, C_4_ = CH_2_]; (**S**) *α*-cedrene [R_1_ = CH_3_; R_2_ = CH_3_; R_3_ = not; R_4_ = CH_3_; C_1_, C_4_ = CH_2_; C_5_ = C_6_]; (**T**) cyperene [R_1_ = H; R_2_ = CH_3_; R_3_ = H; R_4_ = C_2_, C_6_CH_2_(CH_3_)_2_]; (**U**) copaene; (**V**) *α*-copaene; (**X**) *β*-copaene; (**W**) *γ*-elemene; (**Y**) *β*-farnesene; (**Z**) trans-*β*-farnesene; (**A2**) germacrene B [R_1_ = CH_3_; R_2_ = C(CH_3_)_2_; C_6_ = C_7_; C_2_ = C_10_]; (**B2**) germacrene D [R_1_ = CH_2_; C_4_ = C_5_; C_9_ = C_10_]; (**C2**) *α*-guaiene [R_1_ = C(CH_2_)CH_3_]; (**D2**) *β*-guaiene [R_1_ = (CH_3_)_2_]; (**E2**) *γ* -guaiene [R_1_ = CH(CH_3_)_2_; C_6_ = C_7_]; (**F2**) humulene; (**G2**) *α*-humulene; (**H2**) *β*-humulene; (**I2**) ledol; (**J2**) longicyclene; (**K2**) longifolene; (**L2**) Longipinene; (**M2**) *α*-multijugenol [R_1_ = H; R_2_ = OH; A = 4-CH_3_ - hexcycl-3-ene]; (**N2**) t-muurolol; (**O2**) a-muurolene [R_1_ = CH_3_; R_2_ = not; C_7_ = C_8_; A = 4-Me-hexcycl-3-ene]; P2: *γ*-muurolene [R_1_ + R_2_ = CH_2_; A = 4- CH_3_-hexcycl-3-ene]; Q2: caryophyllene oxide; (**R2**) *α*-selinene [R_1_ = H; R_2_ = CH_3_ *cis*]; (**S2**) *β*-selinene [R_1_ = H; R_2_ = CH_3_ *trans*]; (**T2**) *β*-sesquiphellandrene; (**U2**) viridiflorol; (**V2**) *β*-vetivenene; (**X2**) *α*-ylangene.

**Figure 2:**
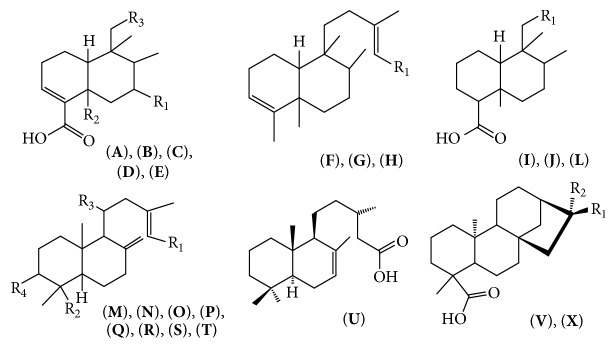
Diterpenes found in copaiba oils. (**A**) Patagonic acid [R_1_ = H; R_2_= CH_3_; R_3_ = furanone]; (**B**) hardwickiic acid [R_1_ = COOH; R_2_ = H; R_3_ = furan]; (**C**) 15,16-epoxy-7*β*-acetoxy-3,13(16),14-clerodatriene-18-oic acid [R_1_ = H; R_2_ = H; R_3_ = furan]; (**D**) 7-hydroxyhardwickiic acid [R_1_ = OH; R_2_ = CH_3_ R_3_ = furan]; (**E**) clerodane-15,18-dioic acid [R_1_ = H; R_2_ = CH_3_; R_3_ = CH(CH_3_)CH_2_COOCH_3_]; (**F**) 3,13-clerodadiene-15-oic acid [R_1_ = COOH]; (**G**) colavenol [R_1_ = CH_2_OH,* trans* C_1_, C_2_]; (**H**) Cis-colavenol [R_1_ = CH_2_OH* cis* C_1_, C_2_]; (**I**) 13-clerodane-15,16-olideo-18-oic acid [R_1_ = furanone]; (**J**) clerodane-15,18-dioic acid [R_1_ = CH_2_(CH_3_)CH_2_CH_2_COOH]; (**L**) clorechinic acid [R_1_ = furan]; (**M**) copaiferolic acid [R_1_ = COOH; R_2_ = OH; R_3_ = H; R_4_ = H]; (**N**) copaiferic acid, [R_1_ = COOH; R_2_ = CH_3_; R_3_ = H; R_4_ = H]; (**O**) 8(17), 13-labdadiene-15-ol [R_1_ = CH_2_OH; R_2_ = CH_3_; R_3_ = H; R_4_ = H]; (**P**) 11-hydroxycopalic acid [R_1_ = COOH; R_2_ = CH_3_; R_3_ = OH; R_4_ = H]; (**Q**) ent-3-hydroxy-labda-8(17),13-diene-15-oic acid [R_1_ = COOH; R_2_ = CH_3_; R_3_ = H; R_4_ = OH]; (**R**) ent-agatic acid [R_1_ = COOH R_2_ = COOH R_3_ = H R_4_ = H]; (**S**) copalic acid [R_1_ = COOH; R_2_ = CH_3_; R_3_ = H; R_4_ = H]; (**T**) 11-acetoxy-copalic acid [R_1_ = COOH; R_2_ = CH_3_; R_3_ = CO_2_CH_3_; R_4_ = H]; (**U**) cativic acid; (**V**) ent-16(*β*)-cauranic-19-oic acid [R_1_ = CH_3_; R_2_ = H]; (**X**) ent-caura-16-ene-19-oic acid [R_1_ and R_2_ = CH_2_]; R_1_ and R_2_ need for two connections, one and one *πσ*.

The biological properties of* Copaifera *spp. have been assigned to the diterpenes and sesquiterpenes [28, 29]. The most common sesquiterpenes were caryophyllene, *α*-copaene, zingiberene, *β*-bisabolene, and bergamotene. The main diterpenes were kaurenoic, hardwickiic, kovalenic, polyalthic, and copalic acids. The diterpene was major metabolite of* Copaifera*. Many phytochemical studies have been conducted with species* Copaifera* and identified several sesquiterpenes (Figure 1) [28–34] and diterpenes clerodanes (Figure 2) [35–40], and labdanes (Figure 2) [34, 39–43].  Table 1 lists all substances identified in copaiba oil and the figures show chemical structures of the major constituents.

This study describes the traditional use of different species of* Copaifera* in the treatment of wounds, such as wound healing and leishmaniasis. It also relates this information to phytochemical studies and evaluation of activity and toxicity.

## 2. *Copaifera*'s Traditional Use in the Treatment of Wounds and Leishmaniasis

The most abundant* Copaifera* species in the Amazon region are* Copaifera officinalis*,* Copaifera reticulata*, and* Copaifera multijuga *Hayne [24]. Many ethnobotanical studies have been shown.

According to Maciel et al. (2002) [44], it is not always possible to identify the origin of this oil (the species that originated it) or the time and place of collection. The* C. multijuga* oil activity and other species vary with the chemical composition of the oil, and this is influenced by the time and place of harvest [10, 44].

*Copaifera* species are used by the population of Barão de Igarape Miri, state of Para, Brazil, for the treatment of poorly healing wounds [11]. The oils of* C. guyanensis*,* C*.* multijuga*, and* C. officinalis* (Table 2) should be applied two times a day on the affected part for healing ulcers and wounds [45] but without excess [46]. For skin and wound problems, topical application of the remedy of one part oil for 5–10 part glycerin oil is still indicated [47].

The anti-inflammatory activity has been related to sesquiterpene, in particular *β*-bisabolene and *β*-caryophyllene. Also, some of the diterpenes from the type of kauranes, clerodanes, and labdanes have been identified in copaiba oil. These can contribute to the anti-inflammatory activity of oil [13, 24, 48, 49].

The study of Pinto (2008) reports the use of* Copaifera sp*. tea for the treatment of wounds (Table 2). Overall, adverse effects of copaiba are dose-related. High doses of the oil can cause gastrointestinal irritation, diarrhea, salivation, and depression of the central nervous system [44]. At a dose of 10 g shown symptoms of intolerance are nausea, vomiting, cramps, diarrhea, and rash [50].

Some studies evaluating the toxicity of copaiba have already been performed. Pregnant rats were subjected to treatment with copaiba oil (500, 1000, and 1250 mg/kg orally), and there was no observed embryotoxicity effect at any dose [51].

The hydroethanol extract of leaves from* C. langsdorffii *was subjected to evaluation of genotoxicity by the micronucleus test. The mice were treated and genotoxicity was evaluated in acute treatment (24 and 48 h) and after multiple doses (7, 15, and 21 days). This extract was not genotoxic and increased exposure time and dose did not interfere with this toxicity [52]. The ethanol extract obtained from the pulp of the fruit of* C. langsdorffii *presented antioxidant activity and was not genotoxic [53].

Although the extracts and the copaiba oil showed low cytotoxicity, the kaurenoic acid has been demonstrated to be toxic. Continuous exposure of sea urchin embryos* (Lytechinus variegatus)* to kaurenoic acid, starting immediately after the fertilization, progressively inhibited its development (IC_50_ of blastulas: 44.7 mM; IC_50_ of lavae stages: 10 mM). In the cell viability assay, kaurenoic acid (concentration of 78 mM) inhibited the growth of leukemic cells (95%), breast, and colon cancer (45% each). Furthermore, kaurenoic acid induced hemolysis in a dose-dependent manner in rat and human erythrocytes (IC_50_ of 74.0 and 56.4 mM, respectively). These results indicate the cytotoxicity of kaurenoic acid [54].

## 3. *Copaifera*'s Traditional Use Validation

The chemical composition variations of* C. reticulata* and the concentration of the main volatile compounds were identified by gas chromatography-mass spectrometry (GC-MS). Almost 100% were sesquiterpenes constituents with the three major compounds: *β*-caryophyllene, trans-*α*-*β*-bergamotene, and bisabolene. However, there is a high intrapopulation variability in composition and concentration of the sesquiterpenes. However, it was unclear whether environmental, morphometric, and structural factors would affect the composition of oleoresin, although some compounds vary according to soil type, volume of extracted oleoresin, and crown surface [55].

*Leishmania amazonensis* is responsible for most cases of American cutaneous leishmaniasis (ACL) in the Brazilian Amazon. The ACL is a disease of worldwide occurrence, and approximately 95% of cases occur in the Americas, the Mediterranean basin, the Middle East, and Central Asia [56]. In Brazil, from 1990 to 2013, about 18,226 cases of cutaneous leishmaniasis were reported, and over 46% were recorded in the North [57].

The parasite cycle begins in the body after the blood meal, and its infectious form is the metacyclic promastigote. Few hours later, the parasite is phagocytosed and within the macrophages it differs in the amastigote form, which multiplies intensely until its rupture, resulting in the release of these forms that will be phagocytosed by new macrophages in a continuous process, resulting then in hematogenous dissemination to other tissues rich in cells of the mononuclear phagocytic system, such as lymph nodes, liver, spleen, and bone marrow [58].

The evaluation of the leishmanicidal activity of copaiba was carried out mainly in strains of* L. amazonensis*. The leishmanicidal activity of* C. reticulata* in* L. amazonensis* was influenced by the chemical composition of the oil. The sample with lower content of copalic acid and kaurenoic acid is the most active one (Table 3). *β*-Caryophyllene can be considered as a marker compound of leishmanicidal activity [59], probably being in higher concentration in the samples of the oil from Para (Table 3).

In the amastigote forms of* L. chagasi *(Table 3),* C. reticulata* oil had higher activity, while in* L. amazonensis* it has shown higher activity against promastigote forms [15]. The oils obtained from* Copaifera martii*,* Copaifera cearensis*,* Copaifera pauper*,* Copaifera langsdorffii*,* Copaifera multijuga*, and* Copaifera lucens* have been shown to be active against promastigote form of* L. amazonensis* (IC_50_ 10–22 *µ*g/mL). Only the oil from* Copaifera pauper* was not active against leishmania (Table 3) [10, 15].

In general, the isolated substances from the oils of copaiba showed higher activity against the amastigote forms of* L. amazonensis*, except hydroxycopalic acid (Table 3). Diterpene acids (such as pinifolic acid and kaurenoic acid) induced a considerable increase in plasma membrane permeability in the axenic amastigote forms of* L. amazonensis* [17]. That may explain the highest activity against this form of the parasite.

Other studies evaluated the activity of the isolated terpenes from copaiba in different forms of* Trypanosoma cruzi*. Amastigotes were more sensitive to the presence of different compounds from* Copaifera*. [60]. Similarly, the amastigote forms were more susceptible to the substances isolated from the copaiba oil (Table 3).

Exposing the parasites to the hydroxycopalic acid causes structural alterations as changes in the cell shape, flagellar membrane, and rupture of the plasmatic membrane. The loss of cellular material, abnormal condensation of chromatin, and intense exocytic activity in the region of the flagellar pocket is the most significant observation. The changes of the mitochondrial swelling and the appearance of concentric membranes in the interior of the organelles are found [17].

From the tested compounds, the majority did not lead to lipid peroxidation that occurs in the presence of reactive oxygen species (ROS) and may be related to the mitochondrial damage or inhibition of the detoxification system. The lipid peroxidation reaction takes place in the presence of reactive oxygen species (ROS) and may be associated with mitochondrial damage or inhibition of detoxification system [17].

When the cytotoxicity (IC_50_) and the activity against promastigote and amastigote forms (IC_50_) of the oil and isolated substances are related, selectivity is observed, meaning that the cytotoxic concentration 50% is higher than the inhibitory 50% (Table 3). The cytotoxicity of terpenes obtained from the copaiba oil was evaluated using a culture of mammal cells LLCMK2 and erythrocytes. Regarding the hemolytic potential, low toxicity was identified in the majority of terpenes, with 50% of hemolysis in concentrations above 400 *μ*M. The pinifolic acid was the least hemolytic, causing around 8% of hemolysis in concentrations beyond 1500 *μ*M. The copalic acid and the 3*β*-hydroxycopalic acid were the most aggressive to the erythrocytes, causing lysis in 50% in doses bellow 200 *μ*M [60].

Concerning the cytotoxicity to the nucleated cells, the terpenes were considered moderately toxic. The very low potential of *β*-caryophyllene against these cells, with a IC_50_ above 1700 *μ*M, is highlighted while for the copalic acid and the 3*β*-hydroxycopalic acid IC_50_ was 39.1 and 31.2 *μ*M, respectively [60]. In summary, the caryophyllene had low toxicity, and caryophyllene oxide showed activity against amastigote forms; however, studies of genotoxicity and mutagenicity and in vivo studies should be performed.

The association with *β*-caryophyllene and copalic acid showed a synergic effect to the activity against* Trypanosoma cruzi* [60]. Unfortunately, the evaluation of the synergic effect of these substances against amastigote forms of* L. amazonensis* has not yet been performed.

The acute infection of cutaneous leishmaniasis (LCL) is characterized by the presence of an inflammatory profile with an increase of T-helper 1 (Th1) response and an increase in the population of cells responsible for the production of Interleukin 12 (IL-12) and interferon gamma (IFN-y) [61]. IL-12 is a cytokine primarily released by macrophages and is known for its important role in the immunopathology of leishmania since both recruit T cells and natural killer (NK) cells [62]. This type of response helps reduce the parasite load resulting in the elimination of infection [63]. IL-1b is also a characteristic cytokine of an inflammatory response. This cytokine results from the activation of the caspase-1-dependent inflammasome and is critical for the control of infection, as this pathway actives iNOS and consequently the nitric oxide [64]. In the case of chronic lesions, a high load is associated with the presence of cells production of IL-10, favoring the recruitment of regulatory T cells (Treg) and, as a consequence, an anergic T cell response (no antigen-specific response). The profile in the chronic infection is characterized by the presence of cells with the Th2 profile and an increased production of cytokines such as IL-2, IL-4, IL-6, IL-10, and transformation growth factor beta (TGF-b) leading to an anti-inflammatory response [65–70] and promoting spread of disease to other locations [70].

The immunomodulatory effect of natural products in Peripheral Blood Mononuclear Cells (PBMCs) may occur mainly through its action in the monocytes and their receptors that recognize pathogens. After cell activation, adaptor proteins can activate the transcription of the nuclear factor *κ*B (NF-*κ*B), which will lead to the expression of genes of cytokines, chemokines, and antimicrobial peptides and of costimulatory molecules [71, 72]. Also, the effects of* Copaifera reticulata*,* Copaifera duckey*, and* Copaifera multijuga* in monocytes and the viability and the production of pro- and anti-inflammatory cytokines (TNF-*α* and IL-10, respectively) were performed. In all concentrations (5, 10, and 20 *μ*g/mL) the oils did not affect the cell viability (>85%) and the production of TNF-*α* induced by LPS was maintained. However, the oils reduced the production of IL-10 significantly [72]. The interference in the production of cytokines suggested that the copaiba oil, probably, interferes in the chronical immune response of leishmaniasis.

Aside from the antiparasitic activity, another important question to be analyzed is whether the use of copaiba oil has a healing effect on the ulcers. The action of the copaiba oil in the cicatrization processes has been confirmed in some studies; further studies to prove with more accuracy the influence of the copaiba oil in the healing process and its toxic effects are necessary [73].

The oil resin activity of* C. langsdorffii* was evaluated in an incision wound model in rats and the contraction of the excised wounds was observed in addition to measuring the tensile strength in wound healing. Topical application of oleoresin accelerated wound contraction indicating a beneficial effect of the oil resin of* C. langsdorffii* in wound healing, thus justifying its traditional use for the treatment of wounds [9].

The cicatrization processes of cutaneous ulcers on the backs of adult male rats were compared in a histological study, where during 15 days topical applications of the copaiba oil were performed twice a day. It resulted in cicatrizations similar to the initial and final periods of the treatment and in the intermediaries of 7 days showed a complete epithelization in the cutaneous lesions, even though the copaiba oil has a slow capacity of repairing connective tissues [74].

## 4. Conclusions

Copaiba oil and its isolated terpenes have antiparasitic activity, being more promising against amastigote forms of* L. amazonensis*. This activity is probably related to the alterations in the membrane and mitochondria. This oil showed low cytotoxicity and genotoxicity. Besides that, it seems that this oil may interfere with the immune response to the infection and has ulcer healing effect. In summary, copaiba oil is promising as a leishmanicidal agent.

## Figures and Tables

**Table 1:** Terpenes present in *Copaifera.*

Sesquiterpenes	Diterpenes	
	Clerodanes	Labdanes
Alloaromadendrene, ar-curcumene, *α*-bergamotene, *β*-bergamotene, ar-curcumene, bicyclogermacrene, *β*-bisabolene, *β*-bisabolol, cadalene, cadinene, *α*-cadinene, *δ*-cadinene, *γ*-cadinene, *α*-cadinol, calamenene, caryophyllene, *β*-caryophyllene, *α*-caryophyllenol, cedrol, *α*-cedrene, cyperene, copaene, *α*-copaene, *β*-copaene, *γ*-elemene, *β*-farnesene, *trans*-*β*-farnesene, germacrene B, germacrene D, *α*-guaiene, *β*-guaiene, y-guaiene, guaiol, humulene, *α*-humulene, *β*-humulene, *γ*-humulene, ledol, longiciyclene, *α*-multijugenol, t-muurolol, *α*-muurolene, *γ*-muurolene, caryophyllene oxide, *α*-selinene	3,13-clerodadiene-15,16-olide-18-oic acid 3-clerodene-15,18-dioic acid 13-clerodene-15,16-olide-18-oic acid 3,13-clerodadiene-15-oic acid 3,13-clerodadien-15-ol *ent*-15,16-epoxy-7*β*-hydroxy-3,13(16),14-clerodatrien-18-oic acid *ent*-(19a)-3,13-clerodadien-15-ol *ent*-*neo*-4(18), 13-clerodadien-15-ol clerodene-15,18-dioic acid *ent*-15,16-epoxy-13(16),14-clerodadien-18-oic acid *ent*-15,16-epoxy-3,13(16),14-clerodatrien-18-oic acid (+)-7*β-*Acetoxy*-*15,16*-*epoxy*-*3,13(16*),*14*-*clerodatrien*-*18*-*oic acid	*ent*-3-hydroxy-labda-8(17),13-dien-15-oic acid *ent*-8(17),13-labdadien-15,19-dioic acid *ent*-8(17)-labden-15-oic acid *ent*-8(17)-labden-15,18-dioic acid *ent*-15,16-epoxy-8(17),13(16),14-labdatrien-18-oic acid 18-hydroxy-8(17),13-labdadien-15-oic acid 8(17), 13E-labdadien-15-oic acid (13S)-7-labden-15-oic acid 3*β*-hydroxy-15,16-dinorlabda-8(17)-en-13-one 8(17),13-labdadien-15-ol

**Table 2:** Major metabolites of *Copaifera* used in traditional Amazon medicine.

Species	Medical use	Part	Major metabolites	References
*Copaifera* sp.	Treatment of injury/wound	Oil; tea	Copalic acid; kaurenoic acid; hardwickiic acid	Pinto, 2008 [11]; Santos et al., 2008 [10]

*C. multijuga*	Healing	Stalk (decoction)	Copalic acid; hardwickiic acid	Center of the Workers of the Amazon (CTA), 1996 [12]; Santos et al., 2008 [10]

*C. guyanensis*	Healing	Stalk: oil	Kaur-16-en-19-oic acid; polyalthic acid; hardwickiic acid	Veiga Jr. and Pinto, 2002 [13]; Cascon and Gilbert, 2000 [14]

*C. officinalis* (Jacq.) L.	Healing and leishmaniasis	Stalk: oil	—	Center of the Workers of the Amazon (CTA), 1996 [12]

*C. reticulata*	Healing	Oil: oil	Copalic acid; kaurenoic acid	Santo**s** et al., 2008 [10]

**Table 3:** Antileishmanial activity and cytotoxicity of *Copaifera* and terpenes present in this genre.

Species	Promastigote (IC_50_-*µ*g/mL)	Amastigote (IC_50_-*µ*g/mL)	Cytotoxicity (IC_50_-*µ*g/mL)/IS	Chemical composition	Reference
*Copaifera reticulate*	7.88	0.52	ND		Rondon et al., 2012 [15]

*Copaifera reticulate* (Pará)	5.0 ± 0.8	20.0	40.0/8.0 e 2.0	Copalic (2.4%); kaurenoic (3.9%) acid	Santos et al., 2008 [10]

*Copaifera reticulata* (Acre)	22.0 ± 0.0	ND	ND	Copalic (7.7%), kaurenoic (7.5%), hardwickiic (6.9%) acid	Santos et al., 2008 [10]

*Copaifera martii*	14.0 ± 0.9	ND	ND	Kaurenoic (7.9%); kovalenic (29.0%) acid	Santos et al., 2008 [10]

*Copaifera cearensis*	18.0 ± 0.0	ND	ND	Hardwickiic (6.2%); copalic (2.1%) acid	Santos et al. 2008 [10]

*Copaifera paupera*	11.0 ± 0.4	ND	ND	Copalic (6.1%); kaurenoic (13.3%)	Santos et al., 2008 [10]

*Copaifera langsdorffii*	20.0 ± 0.8	ND	ND	Copalic (5.6%); kaurenoic (44.3%); hardwickiic (8.2%) acid	Santos et al., 2008 [10]

*Copaifera officinalis*	20.0 ± 0.4	ND	ND	Copalic (13.9%); hardwickiic (30.7%) acid	Santos et al., 2008 [10]

*Copaifera multijuga*	10.0 ± 0.8	ND	ND	Copalic (6.2%)	Santos et al., 2008 [10]

*Copaifera lucens*	20.0 ± 0.9	ND	ND	Copalic (11.1%); polyalthic (69.8%) acid	Santos et al., 2008[10]

*Copaifera paupera *(Herzog) Dwyer	>100	>100			Estevez et al., 2007 [16]

Kaurenoic acid	28.0 ± 0.7	3.5 ± 0.5	140.0 ± 17.0/40.0	—	dos Santos et al., 2013 [17]

Hydroxycopalic acid	2.5 ± 0.4	18.0 ± 1.5	40.0 ± 2.4/2.2	—	dos Santos et al., 2013 [17]

Polyalthic acid	35.0 ± 2.0	15.0 ± 1.0	>500/>33.3	—	dos Santos et al., 2013 [17]

Pinifolic acid	70.0 ± 8.0	4.0 ± 0.4	>500/>125.0	—	dos Santos et al., 2013 [17]

Caryophyllene oxide		2.9	85.0	—	Soares et al., 2013 [59]

Sesquiterpenes		2.3	92.4	—	Soares et al., 2013 [59]

Amphotericin B	0.06 ± 0.0	0.23 ± 0.0	ND	ND	dos Santos et al., 2013 [17]
